# Genetic and biochemical studies in Argentinean patients with variegate porphyria

**DOI:** 10.1186/1471-2350-9-54

**Published:** 2008-06-20

**Authors:** María V Rossetti, Bárbara X Granata, Jimena Giudice, Victoria E Parera, Alcira Batlle

**Affiliations:** 1 Centro de Investigaciones sobre Porfirinas y Porfirias, Hospital de Clínicas, CONICET, Buenos Aires, Argentina; 2 Facultad de Ciencias Exactas y Naturales, University of Buenos Aires, Argentina

## Abstract

**Background:**

A partial deficiency in Protoporphyrinogen oxidase (PPOX) produces the mixed disorder Variegate Porphyria (VP), the second acute porphyria more frequent in Argentina. Identification of patients with an overt VP is absolutely important because treatment depends on an accurate diagnosis but more critical is the identification of asymptomatic relatives to avoid acute attacks which may progress to death.

**Methods:**

We have studied at molecular level 18 new Argentinean patients biochemically diagnosed as VP. PPOX gene was amplified in one or in twelve PCR reactions. All coding exons, flanking intronic and promoter regions were manual or automatically sequenced. For RT-PCR studies RNA was retrotranscripted, amplified and sequenced. PPOX activity in those families carrying a new and uncharacterized mutation was performed.

**Results:**

All affected individuals harboured mutations in heterozygous state. Nine novel mutations and 3 already reported mutations were identified. Six of the novel mutations were single nucleotide substitutions, 2 were small deletions and one a small insertion. Three single nucleotide substitutions and the insertion were at exon-intron boundaries. Two of the single nucleotide substitutions, c.471G>A and c.807G>A and the insertion (c.388+3insT) were close to the splice donor sites in exons 5, 7 and intron 4 respectively. The other single nucleotide substitution was a transversion in the last base of intron 7, g.3912G>C (c.808-1G>C) so altering the consensus acceptor splice site. However, only in the first case the abnormal band showing the skipping of exon 5 was detected. The other single nucleotide substitutions were transversions: c.101A>T, c.995G>C and c.670 T>G that result in p.E34V, p.G332A and W224G aminoacid substitutions in exons 3, 10 and 7 respectively. Activity measurements indicate that these mutations reduced about 50% PPOX activity and also that they co-segregate with this reduced activity value. Two frameshift mutations, c.133delT and c.925delA, were detected in exons 3 and 9 respectively. The first leads to an early termination signal 22 codons downstream (p.S45fsX67) and the second leads to a stop codon 5 codons downstream (p.I309fsX314). One reported mutation was a missense mutation (p.G232R) and 2 were frameshift mutations: c.1082insC and 1043insT. The last mutation was detected in six new apparently unrelated Argentinean families.

**Conclusion:**

Molecular analysis in available family members revealed 14 individuals who were silent carriers of VP. Molecular techniques represent the most accurate approach to identify unaffected carriers and to provide accurate genetic counselling for asymptomatic individuals. The initial screening includes the insertion search.

## Background

**Figure 1:**
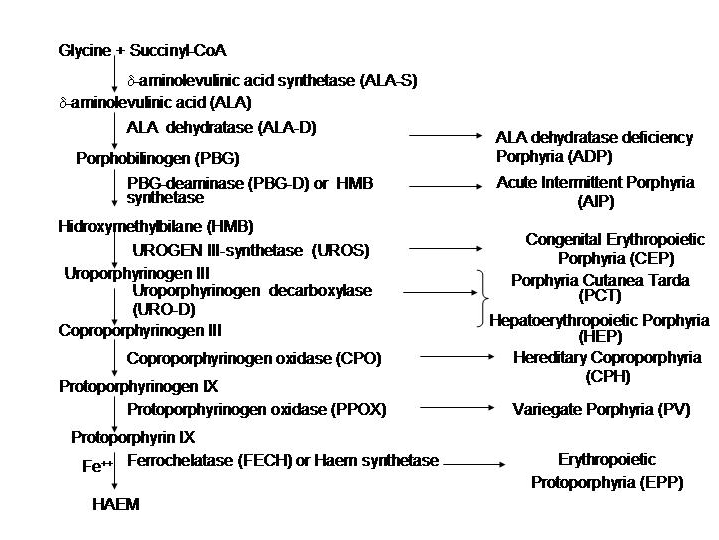
Heme biosynthetic pathway, enzymes involved and associated porphyries.

The hereditary porphyrias are a group of diseases resulting from genetically determined partial deficiencies in one of the heme biosynthetic enzymes. These disorders can be classified on the basis of their clinical manifestations into cutaneous, acute and mixed porphyrias. Variegate Porphyria (VP) (MIN # 176200) is an autosomal dominant disorder associated with a deficiency of the penultimate enzyme of the heme biosynthetic pathway [1-3] the Protoporphyrinogen oxidase [PPOX; EC 1.1.3.4, Genebank accession number X99450.1] which catalyses the six-electron conversion of Protoporphyrinogen IX to Protoporphyrin IX (PROTO IX) (Figure 1)

Patients with VP may manifest a broad spectrum of clinical manifestations characterized by cutaneous photosensitivity and neurological symptoms which can occur separately or together in affected individuals. Cutaneous photosensitivity is characterized by skin fragility, erosions, blisters, millia and pigmentary changes in sun-exposed areas. Neurological symptoms include intermittent attacks of abdominal pain, constipation, vomiting, hypertension, tachycardia, fever and various peripheral and central nervous system manifestations. Acute attacks may frequently result from exposure to diverse porphyrinogenic drugs, alcohol ingestion, reduced calories intake due to fasting or dieting, infections and hormones which stimulate heme synthesis by δ-aminolevulinic acid synthase (ALA-S) induction thereby increasing the production of the porphyrin precursors ALA and porphobilinogen (PBG) [3-5].

Biochemical features of VP include increased biliary excretion of coproporphyrinogen and protoporphyrinogen and their corresponding porphyrins, commonly measured as fecal porphyrins. The standard biochemically tests for VP diagnosis involve quantification and chromatographic separation of fecal porphyrins, urine and plasma porphyrin analysis. Urine ALA and PBG are also measured for confirmation of an acute attack and for exclusion of other forms of Porphyria. In asymptomatic individuals, urine ALA, PBG and porphyrins were within the reference values, however stool porphyrins used to be elevated even in remission [4,6-8].

VP is an autosomal dominant disorder with incomplete penetrance, with heterozygous individuals exhibiting an approximately 50% reduced PPOX activity [1,2]. However, since the first description of a homozygous VP case in 1984 [9] several true homozygous and compound heterozygous cases have been reported [8,10-16].

Identification of patients with an overt VP is important because treatment depends on an accurate diagnosis but more critical is the identification of asymptomatic relatives, because with the proper education about avoiding porphyrinogenic drugs as well as known triggering factors, acute attacks which may progress to death, can be reduced. Although plasma porphyrin index (PPI) is a more sensitive and specific test for VP diagnosis than fecal porphyrin analysis, neither test is sensitive enough for children diagnosis and both are less sensitive in asymptomatic carriers than in symptomatic patients [17]. So, DNA analysis remains the preferred method for the accurate detection of latent carriers of VP trait and thereby facilitates councelling so that precipitating factors can be avoided, as already indicated.

The 5 kb PPOX gene is localized on chromosome 1q2.2-2.3 and contains one non-coding and 12 coding exons [18-20]. Human cDNA encoding PPOX has been cloned and sequenced [21]. Sequence analysis revealed that PPOX consists of 477 aminoacids with a calculated molecular mass of 50.8 kDa. Northern blot analysis revealed the synthesis of a 1.8 Kb mRNA for PPOX [21].

So far, about 130 different mutations were identified in the PPOX gene causing VP [22]. Mutations in the PPOX gene are heterogeneous and most of them are unique to individual families. The only exception is the missense mutation R59W which is present in about 96% of all South African patients with VP due to a well documented founder effect, so it has been identified as the molecular basis for the high prevalence of VP in Afrikaner population [23-26]. The mutation 1329delTACAC has also been reported as a founder mutation in Chilean population [27].

In the first genetic study of the porphyrias in South America we had described 3 new mutations and 2 previously reported in 6 Argentinean patients with VP [28-30]. In this study, in 18 apparently non-related VP patients, we found 4 new splicing defects, 3 new missense mutations and 2 new small deletions. In other two families 2 previously reported mutations were found. In addition, the mutation 1043insT, previously described for 4 Argentinean VP families [28,30] was now found in 6 new unrelated VP families.

## Methods

### Patients

Informed consent was obtained from all patients prior to their inclusion in the study and the study protocol was approved by the Ethical Committee of the Centro de Investigaciones sobre Porfirinas y Porfirias (CIPYP – Hospital de Clínicas, CONICET).

Fourteen female and 4 male apparently unrelated Argentinean patients were studied. All of them had current symptoms of VP and the diagnosis was made on the basis of their clinical history of at least one acute attack and/or typical cutaneous lesions associated with increased excretion of porphyrins in urine and faeces. The chromatographic profile of fecal porphyrins and plasma porphyrin index (PPI) were determined. All of these biochemical parameters were measured in each patient and available relatives by the methodology already described [31]. For PPI determination 1 ml of heparinized fresh blood was centrifuged at 2000 rpm for 10 minutes, 0.3 ml of the plasma were diluted with PBS (1:10) and its fluorescence was spectrofluorometrically determined. PPI was the ratio between the maximum intensity at 626 nm and that of the baseline at the same wavelength [31]. The final diagnosis of the patients was established by genetic studies. The mode of VP presentation (skin lesions alone, acute attacks alone, or both together) was recorded in all symptomatic patients. Unrelatedness was determined by family inquiries, none of the patients were known to be related. All patients and available relatives, their clinical and biochemical symptoms present at the age of diagnosis and the carrier status are shown in Table 1.

**Table 1:** VP Argentinean families: biochemical and molecular data

				**Urine**						
								
**Family**		**Sex**	**Age**	**ALA (mg/24 h)**	**PBG (mg/24 h)**	**PORPH (ug/24 h)**	**Blood IPP**	**Fecal Porph (ug/w)**	**Symp (C/A)**	**Mutation/Defect**
**I**	P	F	35	2.4	2.9	1212	8.80	896	+/-	c.471G>A/del exon5
	M	F	64	1.2	1.1	120	1.21 (619)	70	-/-	c.471G>A/del exon5
	F	M	64	1.4	1.1	77	1.10 (619)	80	-/-	-------
	Si	F	27	1.2	2.4	1975	9.20	371	+/-	c.471G>A/del exon5
**II**	P	F	45	4.8	6.9	1510	9.71	659	+/+	c.807 G>A/r.spl?
**III**	P	F	35	9.2	21.6	4032	7.80	485	+/+	c.808-1G>C/r.spl?
	C	F	28	1.1	0.8	97	1.70	127	-/-	c.808-1G>C/r.spl?
	C	F	33	ND	ND	ND	ND	ND	-/-	c.808-1G>C/r.spl?
**IV**	P	F	40	2.5	4.2	1814	6.50	2078	+/+	c.338+3insT/r.spl?
**V**	P	F	26	1.3	1.6	149	10.40	2411	+/-	c.101A>T/p.E34V
	Si	F	31	1.2	2.1	539	6.00	1208	+/-	c.101A>T/p.E34V
**VI**	P	F	16	8.0	18.5	2619	10.12	ND	-/+	c.101A>T/p.E34V
**VII**	P	F	28	2.0	1.9	948	8.00	428	-/+	c. 995 G>C/pG332A
	F	M	60	1.6	2.0	30	1.12 (619)	78	-/-	c. 995 G>C/pG332A
	D	F	8	0.6	0.9	82	1.10 (619)	85	-/-	-------
	Si	F	25	2.6	2.0	5	2.28	271	-/-	c. 995 G>C/pG332A
	A	F	57	1.7	2.2	52	4.27	52	-/+	c. 995 G>C/pG332A
	C	F	21	0.6	0.9	30	1.24 (619)	79	-/-	-------
	C	F	15	1.2	0.9	40	1.28 (619)	85	-/-	c. 995 G>C/pG332A
	A	F	89	ND	ND	ND	ND	ND	-/-	-------
**VIII**	P	F	40	2.4	4.2	874	5.50	1030	+/+	c.670T>G/p.W224R
	D	F	17	1.4	1.3	2	1.30 (619)	ND	-/-	-------
	D	F	15	0.9	1.4	34	1.28 (619)	ND	-/-	c.670T>G/p.W224R
	A	F	45	2.5	2.3	450	11.77	893	+/+	c.670T>G/p.W224R
	C	F	23	1.4	1.5	25	1.06 (619)	307	-/-	-------
**IX**	P	M	49	1.8	1.8	2291	10.00	2102	+/-	c.133delT/S45fsX67
**X**	P	F	28	1.4	2.6	330	13.5	688	+/+	c.925delA/p.I309fsX314
**XI**	P	F	37	1.2	3.2	801	12.6	1314	-/+	C.694 G>C/G232R
**XII**	P	M	29	6.5	7.7	765	4.60	250	-/+	c.1082insC/p.S359fsX377
	Si	F	35	3.6	3.2	742	4.20	230	-/+	c.1082insC/p.S359fsX377
	D	F	16	0.5	1.4	25	1.30	ND	-/-	-------
	Ni	F	18	1.1	2.1	158	1.30 (616)	ND	-/-	c.1082insC/p.S359fsX377
**XII**	P	F	38	1.6	2.7	985	5.50	2913	+/+	1043InsT/Y348fsX349
**XIV**	P	M	29	2.3	9.0	2375	7.25	1197	+/-	1043InsT/Y348fsX349
**XV**	P	M	24	4.9	3.3	1355	17.50	2141	+/-	1043InsT/Y348fsX349
	B	M	23	ND	ND	ND	2.16	ND	-/-	1043InsT/Y348fsX349
	Si	F	31	ND	ND	ND	1.20 (619)	ND	-/-	-------
**XVI**	P	F	39	12.7	36.3	1202	9.22	936	+/+	1043InsT/Y348fsX349
**XVII**	P	F	28	8.9	14.5	3502	5.30	1739	+/+	1043InsT/Y348fsX349
**XVIII**	P	F	28	5.7	24.0	3527	13.50	ND	-/+	1043InsT/Y348fsX349
**XIX**	P	F	37	6.1	8.7	1321	11.50	964	+/-	1043InsT/Y348fsX349
	D	F	10	1.2	1.5	87	1.25	ND	-/-	1043InsT/Y348fsX349
	D	F	13	1.4	1.1	65	1.23	ND	-/-	-------
	So	M	6	1.0	ND	64	1.30 (618)	ND	-/-	-------
	Si	F	34	ND	ND	ND	1.18 (618)	ND	-/-	1043InsT/Y348fsX349
**XX**	P	F	33	2.0	4.0	1045	8.80	616	+/-	1043InsT/Y348fsX349
	M	F	59	1.5	3.1	390	7.30	515	+/-	1043InsT/Y348fsX349
	D	F	13	1.3	2.0	96	1.30	92	-/-	-------
	So	M	8	ND	ND	ND	ND	ND	-/-	-------
	B	M	29	ND	ND	ND	ND	ND	-/-	-------
**XXI**	P	F	50	1.3	2.3	2364	6.00	1376	+/+	1043InsT/Y348fsX349
**XXII**	P	F	29	1.5	3.7	317	11.20	1329	+/-	1043InsT/Y348fsX349
	M	F	60	1.2	2.0	26	1.25 (619)	36	-/-	-------
	D	F	17	ND	ND	ND	ND	ND	-/-	1043InsT/Y348fsX349
	D	F	16	ND	ND	ND	ND	ND	-/-	-------
	Si	F	22	1.4	2.0	67	1.25 (619)	ND	-/-	1043InsT/Y348fsX349
	Si	F	35	1.1	1.5	38	1.06 (619)	ND	-/-	-------
	Si	F	35	3.2	2.4	529	1.60 (619)	548	+/-	1043InsT/Y348fsX349
	Ni	F	15	ND	ND	ND	5.33	ND	-/-	1043InsT/Y348fsX349
	Ni	F	10	ND	ND	ND	1.90	ND	-/-	1043InsT/Y348fsX349
	Ni	F	15	ND	ND	ND	1.28 (619)	ND	-/-	-------
	C	F	42	2.2	1.9	47.5	1.25	46	-/-	-------
**XXIII**	P	F	24	6.4	8.2	1108	7.10	1529	+/+	c.503G>A/p.R168H
	So	M	18	1.1	1.2	35	1.15 (619)	ND	-/-	c.503G>A/p.R168H
	So	M	16	2.0	1.5	31	1.02 (619)	ND	-/-	-------
	So	M	19	0.9	0.6	86	120	ND	-/-	c.503G>A/p.R168H
	Si	F	35	ND	ND	ND	3.15	1281	+/-	c.503G>A/p.R168H
**XXIV**	P	F	4	2.8	6.2	488	7.00	1705	+/-	C.745delG/V251fsX272
	M	F	30	1.9	2.9	65	3.20	563	+/-	C.745delG/V251fsX272
	A	F	28	3.0	2.0	114	5.33	2491	+/-	C.745delG/V251fsX272
**XXV**	P	F	27	3.1	4.0	870	5.40	1592	+/-	c.532T>G/p.L178V
**XXVI**	P	F	39	4.4	6.2	307	5.33	1214	+/-	c.317A>C/p.H106P

Clinical and biochemical symptoms at the age of diagnosis, as well as the presence or absence of mutation in the PPOX gene, for all the Argentinean VP patients and the available relatives studied at molecular level are shown. Methodology was as described by Batlle et al, 1997 (30). Normal values were: ALA: 2–4 mg/24 h; PBG: 1–2 mg/24 h; urinary porphyrins: up to 250 μg/24 h; faecal porphyrins: up to 130 μg/dw; PPI: up to 1.30 at λ = 618. P: proband, M: mother, F: father, Si: sister, B: brother, D: daughter, So: son, A: aunt, C: cousin, Ni: niece. Probands of the families XIX to XXVI have been previously studied. All families that carried the 1043insT (families to XIII to XXI) are shown.

sAcute symptoms included abdominal pain, paresthesia, muscle weakness, paralysis and/or data of at least one acute attack. Cutaneous symptoms included blisters, erosions, scaring in sun exposed areas and hyperthricosis. Probands of the families XII, XIII and XVI came first with only cutaneous symptoms but they have also clinical data of neurological manifestations.

### Protoporphyrinogen oxidase activity

PPOX activity was determined following the methodologies described by Brenner and Bloomer [1] and Deybach et al [2].

### Identification of mutations

#### DNA isolation and PPOX gene amplification

Genomic DNA was extracted from peripheral blood leukocytes using the GFX Genomic Blood DNA Purification Kit (Amersham) according to the manufacture's instructions. Exons 1–13 along with 50–100 bp of their flanking regions, intronic and promoter regions of the PPOX gene of each symptomatic VP patient were amplified in one or eleven different PCR reactions by means of the primers and conditions listed in Table II. Standard reaction mixture contained in a final volume of 50 μl: buffer 1×, dNTP's 200 μM, TAQ DNA polymerase (Recombinant or Platinum High Fidelity, Invitrogen), primers 0, 5 μM and variable amounts of Mg^++ ^according to the fragment to be amplified as it indicated in Table II. PCR amplified double stranded DNA was purified either using the QIAQuick PCR purification kit (QIAGEN) or from agarose gels by means of Nucleospin Extract kit (Machery-Nagel) or S.N.AP™ Gel Purification Kit (Invitrogen).

#### Sequencing analysis

Mutations screening was done by manual sequencing employing the Amplicycle Sequencing kit (Applied Biosystems, Roche) or fluorescent automated sequencing in the forward and reverse directions. In the first case the products were analysed in a 8% polyacrylamide gel in the presence of 6 M UREA. Sequencing primers were chosen to include 50–100 bp of flanking regions and sequences were analysed by visual inspection comparing with the normal sequence [20,21]. All mutations were confirmed by sequencing both DNA strands of at least two different PCR products. To validate the new mutations their absence in 50 control individuals by manual or automated sequencing has been performed.

#### RNA Analysis

RNA was isolated using the RiboPure™ Blood kit (Ambion) and was reverse transcribed in a final volume of 20 μl. To 1 μg of denaturated RNA, 1× first strand buffer, 300 μM each dNTP, 0,025 μg oligo dT, 10 μM DDT and 400 units of murine retrotranscriptase (Invitrogen) were added and RT was performed at 37°C for 1 hour with the corresponding primers and the protocol according to the fragment to be amplified depending on the splicing mutation found (Table 2).

**Table 2:** Primers and conditions used for PPOX gene and cDNA amplification

	**Amplified region (exon)**	**Amplified Region (intron)**	**MW (pb)**	**Sequence (5' to 3')**	**Annealing (°C)**	**Mg^+2 ^(mM)**
**I**	2, 3, 4	2, 3	758	F: GCTTCTGGAGCGCAGGTTGTCC R: AAGGCATATGAGGATGAGGGCA	60	1.6
**II**	5, 6	5	680	F: AGGTATGTCAGGAGCTTCCCCC R: CCCTCACTTTGGCAGTACTTAA	60	1.6
**III**	7, 8	7	852	F: TGCTGGGATTACAGGTGT R: AGCTTTTGCTTCTCACTGGTAGG	62	2.5
**IV**	9	-----------	321	F: GATTACAGGTGTGAGCCACCA R: CCTACCAGTGAGAAGCAAAAGCT	60	2
**V**	10, 11,12, 13	10,11,12 13	859	F: GCCCTTTCCTTCTGACGCATG R: GCCAGACCAAGCCAAGCCAAGC	62	2.5
**VI**	1	1	440	F: CCAAGTCCCGCCAATCCAGAT R: GGACAACCTGCGCTCCAGAAGC	60	5
**VII**	Promotor	-----------	705	F: AGGTGATAGAGAACTGGCCCAA R: CAGCCTTTTCGGTCTCTCCTA	63	3
**VIII**	------------	4	786	F: TCTGAGCTTGGCTTGGATTC R: CTCTGCAGCTGGTTTAGG	60	2
**IX**	------------	6	528	F: GCTTTCCCAGTCTCTTCC R: AAGGCCTGGCGAATGAGT	60	2
**X**	------------	8	313	F: ATTCTCATTTTCTGGGTCTCTC R: TCAGCAGGGAGCAGCTCACTG	60	2
**XI**	------------	9	535	F: CTGAGTGCCATCACTGCA R: GCTCAGGGAAAGCAACTG	60	2
**XII**	Gen	All	5,500	F: AGAGAACTGGCCCAAAATTGGAGT R: CAGACCAAGCCAAGCCAAGCAATT	60	2
**4–9**	From nt 226 (exon 4) to nt 893 (exon 9).		668	F: TCTGAGCTTGGCTTGGATTC R: GTCACTCGACGAGGGACGACT	60	3
**6–9**	From nt 478 (exon 6) to nt 893 (exon 9).		416	F: TCTCTAGCCATGGACAGTCT R: GTCACTCGACGAGGGACGACT	60	6

All primers were developed in the course of this study. Primers I to XII were used for DNA amplification and primers 4–9 and 6–9 were used for cDNA amplification.

#### Analysis of missense mutations

The role of the aminoacids involved in the missense mutations in the crystallographic structure of the PPOX protein was evaluated according Koch et al [32] and the deleterious consequences of their replacement for the mutant residues were analised by Swiss PDBViewer program [33].

#### Numbering system

Exonic nucleotides were numbered from the first nucleotide of the translation-initiation codon according to the cDNA sequence derived from PPOX genomic sequence while promoter and intronic nucleotides were numbered according to the complete PPOX genomic sequence [Genebank asccession number X99450.1].

#### Databases

The Human Gene Mutation Database [22] was used for information about reported mutations in the PPOX gene.

## Results

Sequencing analysis of the coding regions and their flanking regions of the DNA from 18 unrelated new patients with VP revealed 9 new mutations and 3 previously identified mutations (Table 1). From the 9 novel mutations 6 were single nucleotide substitutions, 2 were single nucleotide deletions and one was a small insertion.

**Figure 2:**
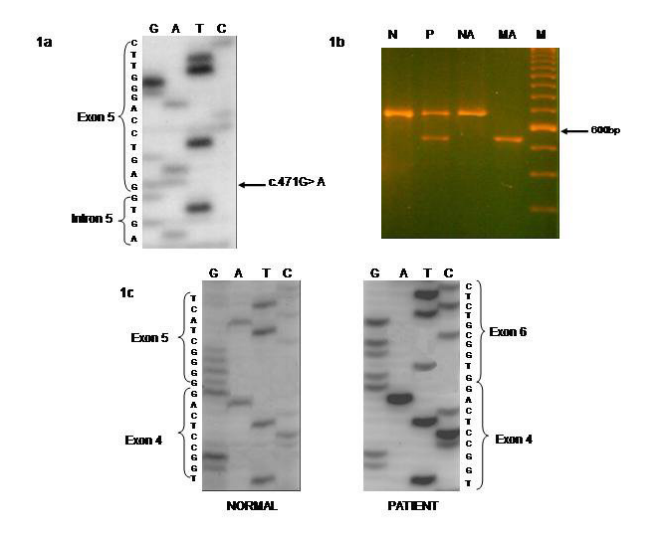
**a)** c.471 G> A mutation in the last base of exon 5; **b)** 4% agarose gel of normal and mutant alleles of the cDNA from the patient showing an extra band of about 530 bp. N = Normal, P: patient; NA: normal allele; MA: mutant allele; M: marker 100 bp; **c)** Sequencing gels for the normal and mutant patient cDNA showing the skipping of exon 5.

**Figure 3:**
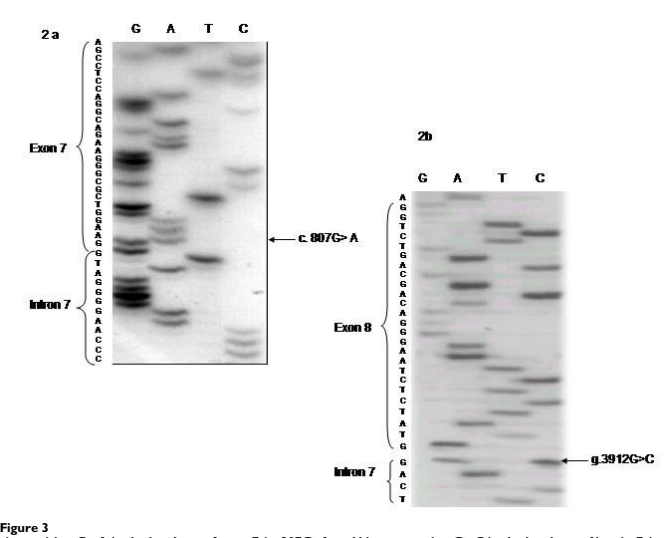
a) transition G> A in the last base of exon 7 (c: 807G>A and b) transversion G> C in the last base of intrón 7 (c. 808-1 G>C).

**Figure 4:**
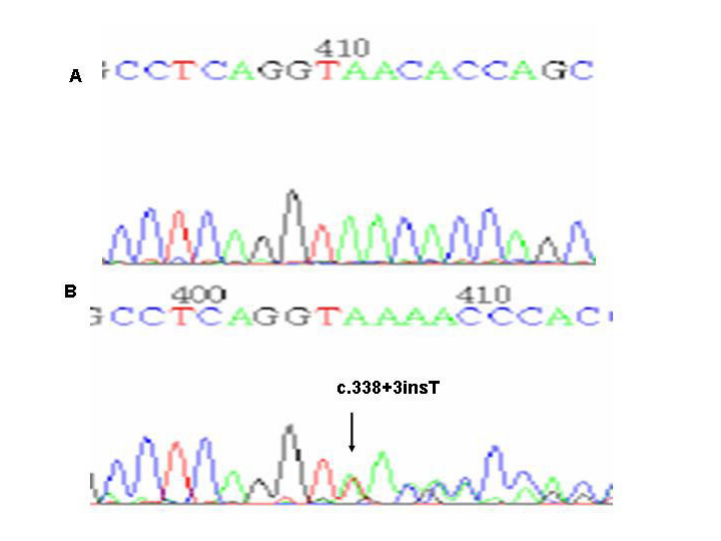
a) Normal sequence and b) Insertion of a T at the nucleotide position 338+3.

Three single nucleotide substitutions and the small new insertion were at exon-intron boundaries regions. Two single nucleotide substitutions (Probands I and II) were G to A transitions in the last bases of exon 5 (c.471G>A) (Figure 2a) and exon 7 (c.807 G>A) (Figure 3a) which do not lead to an aminoacid change but were close to the donor consensus splice sites between exon 5 and intron 5 and between exon 7 and intron 7 respectively and so, they might affect splicing. However, although this base is a residue about 70% conserved for splicing [34] and both changes were absent in 50 normal individuals, only for the first case an abnormal band showing the skipping of exon 5 could be detected (Figure 2b,c). The other single nucleotide substitution (Proband III) was a G to C transversion in the last base of intron 7 (g.3912 G>C) (Figure 3b) which destroys the splice acceptor site between intron 7 and exon 8 (c 808-1 G>C) and is expected to affect normal splicing. Although this is a 100% conserved residue for splicing [32] and this change was absent in 50 control subjects, RT-PCR studies again failed to demonstrate an abnormal band in the RNA's patient. However in both of these cases the value of PPOX activity was about 40% of normal value (Table 3). The other mutation which is very likely to affect the mRNA splicing was a T insertion in intron 4 (g.1742insT), in the boundary of splice donor site between exon 4 and intron 4 (c.338+3insT) (Figure 4). This was the only change found in this patient (Proband IV) and it was also absent in 50 controls. Moreover, activity enzyme in this patient was about 40% of the control (Table 3)

**Table 3:** Protoporphyrinogen oxidase activity

Family	Patient	Total activity nmoles PROTO/mg protein/h	PPOX activity (%)	Mutation/Defect
II	P	16.28	43	c.807 G>A/r.spl?
III	P	13.15	39.55	c.808-1G>C/r.spl?
	C	15.58	46.85	c.808-1G>C/r.spl?
	C	16.85	50.67	c.808-1G>C/r.spl?
IV	P	14.35	42.56	c.338+3insT/r.spl?
V	P	18.48	55.58	c.101A>T/p.E34V
VII	P	15.84	47.64	c. 995 G>C/pG332A
	F	16.89	50.79	c. 995 G>C/pG332A
	C	19.21	57.77	c. 995 G>C/pG332A
VIII	P	16.95	50.98	c.670T>G/p.W224R
	D	18.12	54.19	c.670T>G/p.W224R

PPOX activity was determined as described in Materials and Methods. The values are the mean value of two determinations run in duplicates.

The patients are the probands of the families indicated in Table 1. PPOX activity values of the clinical and biochemical asymptomatic relatives from these families are shown. Normal value: 33.25 ± 6.32 nmoles PROTO/mg protein/h.

P: proband; F: father; D: daughter; C: cousin

**Figure 5:**
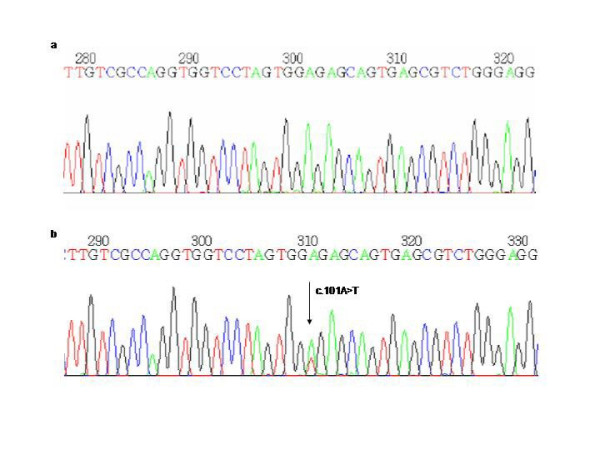
a) Normal sequence and b) Transversion A> T in the nucleotide 101 of exon 3 (E34V).

**Figure 6:**
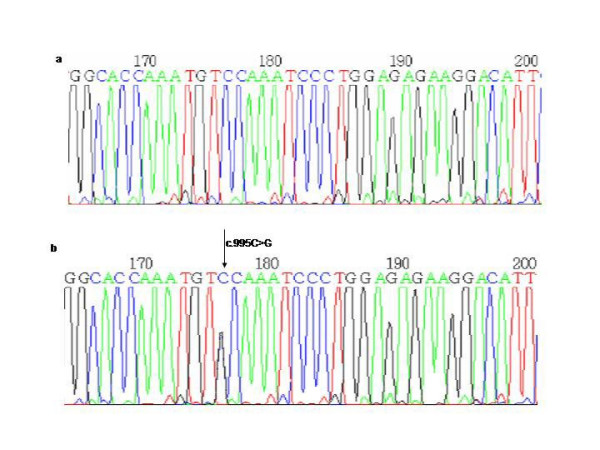
a) Normal sequence and b) Transversion G> C in the nucleotide 995 in exon 10 (G332A).

**Figure 7:**
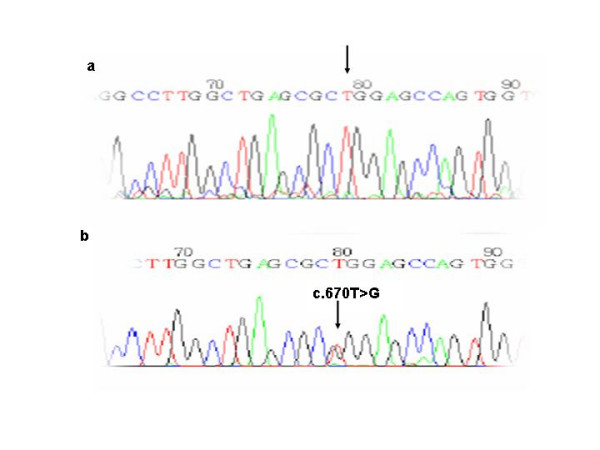
a) Normal sequence and b) Transversion T > G in the nucleotide 670 in exon 7 (T670G).

The other 3 new single nucleotide substitutions were transversions leading to an aminoacid change. One was an A to T substitution at nucleotide position 101 (c.101A>T) (Proband V and VI, Figure 5) which results in a missense mutation that changes a glutamic acid residue to a valine residue in exon 3 (p.E34V). The other was a G to C substitution at nucleotide position 995 (c.995 G>C) (Proband VII, Figure 6) which results in a missense mutation that changes a glycine residue to a alanine residue in exon 10 (p.G332A). The last one was a T to G change at nucleotide position 670 (c.670T>G) leading to a tryptophan to a glycine substitution (p.W224G) (Proband VIII, Figure 7). These mutations were present in the symptomatic relatives studied (Table 1) and they were absent in 50 control individuals. Moreover, PPOX activity studies showed that the enzyme activity was reduced to a value of about 50% in both these patients and their asymptomatic relatives (Table 3).

**Figure 8:**
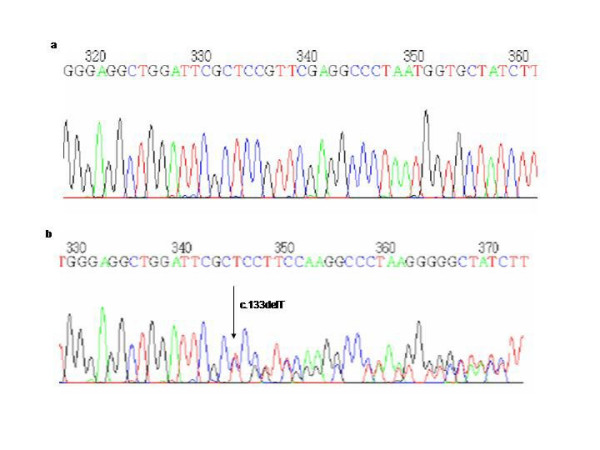
a) Normal sequence and b) Deletion of a T at the nucleotide position 133.

**Figure 9:**
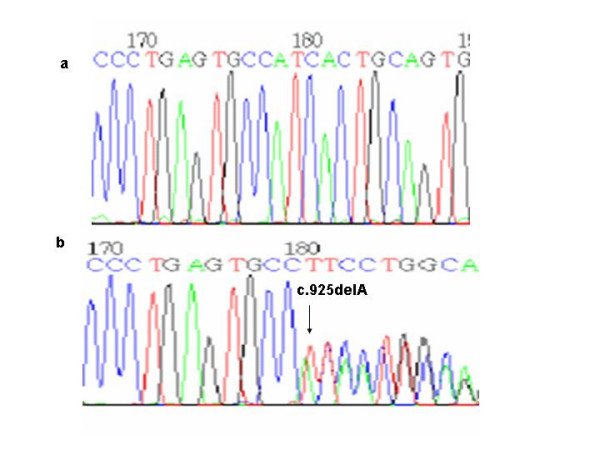
a) Normal sequence and b) Deletion of a A at the nucleotide position 925.

The other 2 new mutations were frameshift mutations which produce an early stop codon. One of them was a timine deletion at nucleotide 133 in exon 3 (c.133delT), (Proband IX, Figure 8) resulting in a frameshift at aminoacid 45 with a premature stop codon 22 codons downstream (p.S45fsX67). The other was an adenine deletion at nucleotide 4215 in exon 9 (Proband X, Figure 9) resulting in a frameshift at aminoacid 309 (c.925delA) with a premature stop codon 5 codons downstream (p.I309fsX314).

In other two families, 2 previously described mutations were found. One was a missense mutation, G232R in exon 7 (Table 1, Proband XI), already published by Deybach et al [35] and D'Amato et al [36]. The other was the insertion 1082insC in exon 10 (Table 1, Proband XII) which results in a frameshift at aminoacid 359 with a premature stop codon 18 codons downstream (p.S359fsX377), so introducing 18 novel amino acids [37-39].

**Figure 10:**
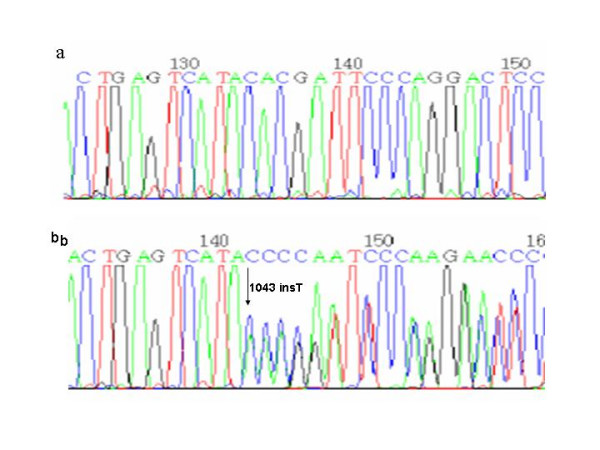
a) Normal sequence and b) Insertion of a T at the nucleotide position 1043.

In addition, six new families (Table 1, Probands XIII to XVIII) carrying the mutation c.1043insT previously described for another four unrelated Argentinean VP patients [28,30] were found (Figure 10). This mutation produces a frameshift at aminoacid 348 and a premature stop codon, 1 codon downstream the inserted nucleotide (p.Y348fsX349).

## Discussion

The elucidation of PPOX nucleotide sequence allowed the detection and characterization of several mutations in this gene associated with VP and since then genetic analysis is the most reliable and accurate methodology for confirmig the diagnosis and most importantly for latent carriers identification.

To date, 72 individuals, belonging to 57 apparently unrelated Argentinean families have been biochemically diagnosed as VP and the analysis of the biochemical data obtained for all these families studied in our Centre showed that the prevalence for this population is about 1:600,000 as previously reported [30], which is very low when compared with others [23-25,37,40].

As it is shown in Table 3, 20 patients (28%) presented only acute symptoms, a value close to that reported by von und zu Fraunberg et al [40] for patients from Finland (29%) before 1980. Moreover, we have 25 patients with only cutaneous symptoms (35%), close to the value also reported by von und zu Fraunberg et al [40] for the Finish population (42%) but at variance with those reported for Western Europe (59%) [37] and for the South African population before (52%) [41] and after 1980 (91%) [42]. It is of note that the percentage of patients with both symptoms (37%) is higher than the value found by Whatley et al [37] and much higher than those found for the Finish [40] and South African populations [41,42] but smaller than our own previous results (50%) [30].

Until now, 52 individuals representing these 26 apparently unrelated Argentinean families have been studied at the molecular level. In Table 1 the clinical symptoms, biochemical findings and the mutation detected for all the new and previously described patients [28-30] as well as all the available relatives are shown. Molecular analysis in those available family members revealed that 15 individuals were silent carriers of VP. This is very important for our VP population since PPI, the most reliable biochemical method to detect VP, was positive in only 5 of them (67%). Besides most of the asymptomatic carriers with a negative plasma peak were under 20 years, as it has been previously found by Hift et al [42]. The number of asymptomatic carriers detected (about 29%), is still much lower than the values reported by others [37,40-42] but unfortunately higher than our own previous results (15%) for the Argentinean population [30]. These results suggest that much work is yet to be done for the Argentinean population to become aware of the vital importance of molecular studies to confirm a putative diagnosis of VP and to detect silent carriers of the porphyria in affected families to prevent the development of the disease.

In this study, in 18 apparently unrelated Argentinean families 9 new mutations have been identified (Table 1). Four of them are expected to inactivate normal splicing sites and so they are likely to affect the normal splicing of the mRNA. Three of them were single nucleotide substitutions, c.471G>A, c.807 G>A and c.808-1G>C and the other was a small insertion. In effect, they might produce the skipping of the exon, retention of all or part of the intron or the activation of a cryptic splicing site [34]. However, only for the c.471G>A mutation (Figure 2a), which affects the donor splicing site for intron 5, the mutant band could be detected indicating exon 5 skipping (Figure 2b, c). For the other 2 mutations, although it is very probably that produce an aberrant splicing because they affect conserved bases for splicing [34], only the normal band could be detected in RT-PCR studies. It would be very likely that in these cases the transcripts resulting from the abnormal splicing were so unstable to be detected and they would be degraded by the so-called nonsense-mediated mRNA decay [43,44].

Other 3 single nucleotide substitutions were transversions leading to missense mutations, c.101A>T, c.670 T>G and c.995G>C in exons 3, 7 and 10 respectively. They were also present in the symptomatic relatives studied and they were absent in 50 healthy controls and thereby it was unlikely to be a common polymorphism. These changes would either give rise to specific aminoacid substitutions at protein level or would affect an exonic splicing enhancer (ESE's) that would exert deleterious effects on the mRNA level by interfering with normal splicing [45-48]. However, the latter possibility seems to be unprobable in these cases. In the p.E34V and p.W224G mutations the glutamic acid and the tryptophan residues are not involved in one of the ESE's sequences predicted for human PPOX [50] In the other case, the p.G332A mutation, although the glycine residue is in fact involved in one of the ESE's sequences predicted for this enzyme (RESCUE-ESE) [49], RT-PCR studies carried out from patient's RNA showed only the normal band suggesting that these base change in this ESE's sequence would not alter the normal splicing of the pre-mRNA.

On the other hand, multiple aminoacid sequence alignment of eukaryotic PPOXs, revealed that mutations p.E34V and p.G332A affect residues that are conserved in mitochondrial PPOX2 from *Nicotiana tabacum and Arabidopsis thaliana *and chloroplast PPOX1 from *Nicotiana tabacum *[32] and also in eucaryotes such as mouse, rat and monkey indicating an important role for both aminoacid residues in the enzyme. In fact, in the crystallographic structure for the enzyme from *Nicotiana tabacum *published by Koch et al [32], E34 residue (E43 for the plant enzyme) lies in the highly conserved FAD-binding domain and the extra carboxyl group is interacting with the cofactor. So, its replacement for a neutral aminoacid would very likely interfere with this interaction [33], so reducing protein activity. On the other hand, G332 (G554 in the plant enzyme) is one of the residues involved in the binding of the inhibitor INH used to model the binding of PROTOgen IX and Proto IX in the crystallographic structure for the enzyme from *Nicotiana tabacum *published by Koch et al [32] and so, this mutation is also likely affecting activity.

The last missense mutation, p.W224G affects a residue which is absent in the crystallographic structure of *Nicotiana Tabacum *[32] but it is conserved in different mammalian such as mouse and monkey. This tryptophan residue seems to play a very important role in the protein activity since it was involved in other two already described mutations causing VP: p.W224X [37] and p.W224R [39]. Recently was also suggested that this aminoacid would likely be in the internal mitochondrial signal targeting [50-52].

Two novel small deletions were detected. One of them was a T deletion, c.133delT in exon 3 introducig 22 novel amino acids before the premature stop codon (p.S45fsX67). The other was an A deletion, c.925delA leading to a stop codon 5 codons downstream (p.I309Sfs314X). Both mutations would produce unstable mRNA's or truncated proteins.

In all cases where the mutation was new and has not been characterized (missense and splicing mutations), enzyme activity values for these patients were such that they reinforced our hypothesis, indicating that these mutations are the disease causing mutations in our VP patients (Table 3). Moreover, PPOX activity values for the clinical and biochemical asymptomatic relatives from these families, indicate that the mutation co-segregates with the 50% enzyme activity (Table 3).

Three already described mutations were also found. One was a missense mutation, p.G232R [34,35]. The other 2 reported mutations were frameshift mutations. One of them was a C insertion in the streach of 6 Cs in exon 10 (c.1077-1082insC), which had been previously described by D'Amato et al [36] and Whatley et al [37]. This same mutation was recently described for the Swiss [38] and Spanish [39] populations. It is of note that while Whatley et al [37] and Lecha et al [39], found this mutation associated with only cutaneous symptoms, the Argentinean family carrying this mutation presents both symptoms as it has been described by Schneider-Yin et al for the Swiss patients [38]. Moreover the E34V mutation was found in two Argentinean unrelated families, one with only cutaneous symptoms (Proband V) and the other with only acute symptoms (Proband VI). These results suggest that no correlation would seem to exist between clinical features and the type of mutation as had been previuosly found by Whatley et al [37]. Moreover the mutation 1043insT, previously described for 4 Argentinean patients [28,30], was found now in 6 new apparently unrelated families.

All new mutations, except E34V as already noted, were restricted to an individual family (Table 1) as it is the case for most of the reported mutations in the PPOX gene. However, there are some mutations that were detected in more than one family. In some cases they seem to represent hot-spot mutations [27,37] but in others a founder effect has been demonstrated [23,25,27,53]. In the present study, from the 27 Argentinean unrelated families studied, 10 families carried the same mutation 1043insT (Table 1), so its prevalence in our population is about 37% (10/27), suggesting that it might represent a common mutation in Argentina. Therefore, the initial screening to elucidate the genetic defect in VP patients from Argentina includes the insertion search. Moreover, as this mutation has not yet been detected in VP patients from other populations, it is very likely that the exclusive occurrence of 1043insT in VP families from Argentine might be due to a founder effect. However, a T insertion one base upstream was recently found for Swiss patients [38] suggesting that perhaps it might represent a hot spot mutation. Haplotype analysis is being carried out to elucidate this question.

## Conclusion

The results presented in this work asses once more that molecular techniques represent the most accurate approach to identify unaffected carriers. This is of vital importance in prepubertal children in whom neither fecal nor plasma porphyrins allow for the identification of the VP carrier status.

Finally, although the number of individuals studied up to now is yet limited, it appears, as it has already been found [37] that no correlation can be established between symptomatology and/or biochemical data and the type of mutation.

## Abreviations

ALA-S: δ-aminolevulinic acid synthase; dNTP: Desoxyribonucleotide phosphate; INH: 4-bromo-3-(5'-carcoxy-4'-chloro-2'-fluoro-phenyl)-1-methyl-5-fluoromethyl-pyrazol; PBG: Porphobilinogen; PCR: Polymerase Chain Reaction; PPI: Plasma Porphyrin Index; PPOX: Protoporphyrinogen oxidase; PROTOgen IX: Protoporphyrinogen IX; PROTO IX: Protoporphyrin IX; RT: Retrotranscription; VP: Variegate Porphyria.

## Competing interests

The authors declare that they have no competing interests.

## Authors' contributions

MVR conceived, designed and supervised the study, carried out the PPOX activity determinations and contributed to writing the manuscript. BXG and JG contribute to the molecular techniques, to some of the PPOX activity determinations and to writing the manuscript. VEP supervised biochemical studies and compiled the clinical data of the patients. AB supervised biochemical studies and porphyria diagnostic and contributed to writing the manuscript. All authors read and approved the final manuscript.

**Table 4:** Clinical features analysis

	N° (%) Subjects from					
	
Symptomatology	Present Study (n: 72)	South Africa^a ^(n: 269)	Souh Africa^b ^(n: 11)	Western Europe ^c ^(n: 103)	Finland^d^	
					
					Before 1980 (n: 34)	After 1980 (n: 20)
Acute	20 (28)	51 (19)	0 (0)	20 (20)	10 (29)	3 (15)
Cutaneous	25 (35)	156 (58)	10 (91)	61 (59)	14 (42)	13 (65)
Both	27 (37)	62 (23)	1 (9)	22 (21)	10 (29)	4 (20)
Asymptomatic	15 (29)	31 (10)	17 (61)	----------	19 (36)	30 (60)
Acute	20 (28)	51 (19)	0 (0)	20 (20)	10 (29)	3 (15)
Cutaneous	25 (35)	156 (58)	10 (91)	61 (59)	14 (42)	13 (65)
Both	27 (37)	62 (23)	1 (9)	22 (21)	10 (29)	4 (20)
Asymptomatic	15 (29)	31 (10)	17 (61)	----------	19 (36)	30 (60)
Acute	20 (28)	51 (19)	0 (0)	20 (20)	10 (29)	3 (15)
Cutaneous	25 (35)	156 (58)	10 (91)	61 (59)	14 (42)	13 (65)
Both	27 (37)	62 (23)	1 (9)	22 (21)	10 (29)	4 (20)
Asymptomatic	15 (29)	31 (10)	17 (61)	----------	19 (36)	30 (60)

The total number of symptomatic subjects were considered

a: Eales et al (1980), b: Hift et al, 2004, c: Whatley et al, 1999, d: von und zu Fraunberg et al, 2000.

## Pre-publication history

The pre-publication history for this paper can be accessed here:


